# Relationship Between eNOS T-786C and G894T Polymorphisms and Colorectal Cancer Susceptibility: A Study in the Algerian Population

**DOI:** 10.3390/ijms27093709

**Published:** 2026-04-22

**Authors:** Fatma Zohra Djaballah-Ider, Ines Gouaref, Ahlem Seghirate, Chafia Touil-Boukoffa, Assia Galleze

**Affiliations:** 1 Laboratory of Cellular and Molecular Biology, Faculty of Biological Sciences, Houari Boumediene University of Sciences and Technology, Algiers 16111, Algeria; 2 Bioenergetics and Intermediary Metabolism Team, Department of Biology and Organism Physiology, Faculty of Biological Sciences, Houari Boumediene University of Sciences and Technology, Algiers 16111, Algeria

**Keywords:** colorectal cancer, eNOS polymorphisms, genetic susceptibility, Algerian population

## Abstract

Colorectal cancer (CRC) is a multifactorial disease influenced by genetic and environmental factors. The endothelial nitric oxide synthase (eNOS) gene, involved in nitric oxide (NO) production, is associated with carcinogenesis. This study aimed to evaluate the association between eNOS −786T>C and G894T polymorphisms and CRC susceptibility in an Algerian population. Genotype and allele frequencies were analyzed, and associations were assessed using odds ratios (ORs) and 95% confidence intervals (CIs). For −786T>C polymorphism, the CC genotype was significantly more frequent in patients than in controls (37.33% vs. 21.67%) and was associated with increased risk of CRC (OR = 2.15, 95% CI: 1.21–3.88, *p* = 0.004), whereas the TT genotype showed a protective effect (OR = 0.41, 95% CI: 0.20–0.81, *p* = 0.005). Regarding the G894T polymorphism, the TT genotype was significantly associated with increased susceptibility to CRC (44.67% vs. 8.33%; OR = 8.88, 95% CI: 4.19–15.40, *p* < 0.001), while the GG genotype was protective (OR = 0.18, 95% CI: 0.10–0.32, *p* < 0.001). Allelic analysis confirmed that the C and T alleles were risk factors. Furthermore, eNOS polymorphisms were significantly associated with tumor location. In conclusion, the eNOS −786T>C and G894T polymorphisms are significantly associated with CRC susceptibility in the Algerian population and could serve as potential genetic biomarkers.

## 1. Introduction

Colorectal cancer (CRC) is one of the most prevalent malignancies worldwide and remains a leading cause of cancer-related mortality. According to recent global cancer statistics, CRC accounts for approximately 10% of all cancer cases, with nearly 1.9 million new cases and more than 900,000 deaths reported annually, ranking it among the three most frequently diagnosed cancers globally. Despite advances in screening, early detection, and therapeutic strategies, CRC continues to represent a major public health burden, largely due to late-stage diagnosis and its increasing incidence among younger populations [1,2]. The pathogenesis of CRC is complex and multifactorial, involving a combination of genetic, environmental, and lifestyle-related factors. At the molecular level, colorectal carcinogenesis is driven by the accumulation of genetic and epigenetic alterations affecting key signaling pathways, including Wnt/β-catenin, KRAS, TP53, and DNA mismatch repair mechanisms, ultimately leading to tumor initiation, progression, and metastasis [3,4,5,6].

Among the genes implicated in CRC development, the endothelial nitric oxide synthase (eNOS) gene has attracted considerable attention. eNOS catalyzes the production of nitric oxide (NO) from L-arginine, a molecule involved in various physiological and pathological processes, including tumor biology [7,8]. Nitric oxide exhibits a dual role in colorectal cancer, exerting both pro-tumorigenic and anti-tumorigenic effects. On one hand, NO may promote tumor growth through the stimulation of angiogenesis; on the other hand, reduced NO levels have been associated with the initiation of carcinogenesis [9,10]. Furthermore, eNOS-derived NO production may increase in response to inflammation and malignancy, suggesting a complex and context-dependent role in CRC progression [11,12,13,14].

The human eNOS gene is located on chromosome 7q35–36 and is characterized by several common polymorphisms, including T-786C, G894T, and intron 4 variable number tandem repeat (VNTR, 4a/b). The T-786C polymorphism involves a thymine-to-cytosine substitution in the promoter region of the gene, where the CC genotype represents the homozygous mutant form, CT the heterozygous genotype, and TT the wild-type genotype [15,16]. The G894T polymorphism is defined by a guanine-to-thymine substitution in exon 7, resulting in the TT homozygous mutant genotype, GT heterozygous genotype, and GG wild-type genotype [17,18,19,20]. Additionally, the intron 4 VNTR (4a/b) polymorphism consists of a variable number of tandem repeats, with aa, ab, and bb representing mutant, heterozygous, and wild-type genotypes, respectively [21,22].

These polymorphisms have been associated with reduced eNOS enzyme activity and decreased nitric oxide production, leading to alterations in endothelial function and tumor-related processes. In particular, these genetic variations may reduce promoter activity and circulating NO levels [23,24]. Moreover, eNOS polymorphisms have been implicated in the development and progression of various cancers [25,26,27,28].

The identification of genetic alterations, such as these gene polymorphisms, could provide valuable molecular biomarkers for the prognosis of colorectal cancer (CRC). Therefore, this study aims to investigate the association between the eNOS T786C and G894T gene polymorphisms and the development and progression of CRC in the Algerian population.

## 2. Results

### 2.1. Patient Characteristics

Clinical data of patients and controls are presented in Table 1. The mean age was 62 ± 13.21 years in patients and 60 ± 10.05 years in controls. There were no significant differences observed between the two groups regarding age (*p* > 0.05), sex (*p* > 0.05), and body mass index (*p* > 0.05).

### 2.2. Genotypic Analysis

**Figure 1:**
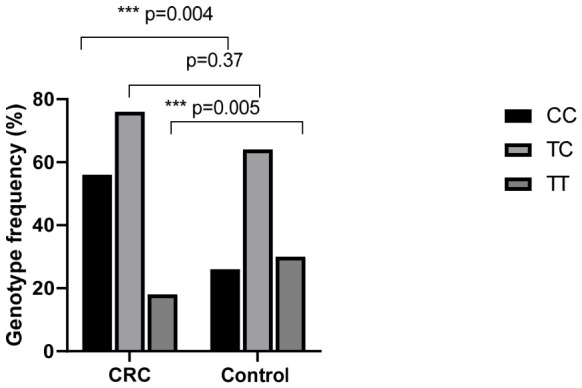
Genotype frequencies of eNOS T-786Cpolymorphism in CRC patients (N = 150) and in the control group (N = 120). The differences between groups were evaluated by the chi-square (χ^2^) test, significances were considered for *p* < 0.05. *** *p* < 0.01.

**Figure 2:**
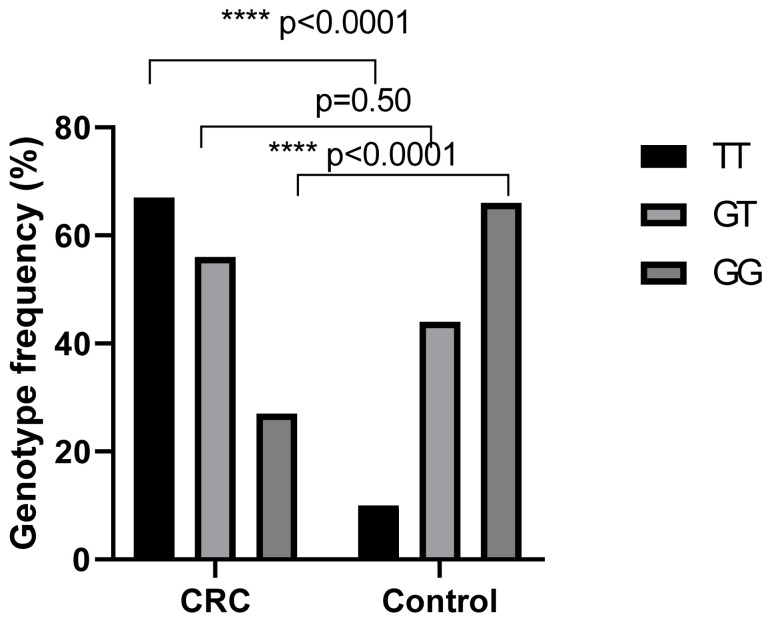
Genotype frequencies of eNOS G894T polymorphism in CRC patients (N = 150) and in the control group (N = 120). The differences between groups were evaluated by the chi-square (χ^2^) test, significances were considered for *p* < 0.05. **** *p* < 0.001.

For each polymorphism, all samples were in Hardy–Weinberg equilibrium (T-786C *p* = 0.49 and G894T *p* = 0.52). The genotype distribution of the eNOS −786T>C and G894T polymorphisms in colorectal cancer patients and controls is shown in Table 2. Regarding the −786T>C polymorphism, the CC genotype was significantly more frequent in patients (37.33%) than in controls (21.67%) and was associated with an increased risk of CRC (OR = 2.15, 95% CI: 1.21–3.88, *p* = 0.004). In contrast, the TT genotype was significantly less frequent in patients (12%) than in controls (25%), suggesting a protective effect against CRC (OR = 0.41, 95% CI: 0.20–0.81, *p* = 0.005). No significant difference was observed for the TC genotype between the two groups (*p* = 0.37) (Figure 1). For the G894T polymorphism, the TT genotype was significantly more frequent in patients (44.67%) than among controls (8.33%), indicating a strong association with CRC susceptibility (OR = 8.88, 95% CI: 4.19–15.40, *p* < 0.001). Conversely, the GG genotype was significantly less frequent in patients (18%) than in controls (55%), suggesting a protective association (OR = 0.18, 95% CI: 0.10–0.32, *p* < 0.001). No statistically significant difference was observed for the GT genotype between patients and controls (*p* = 0.50) (Figure 2).

Furthermore, the C allele of the −786T>C polymorphism was more frequent in CRC patients than in controls (62.7% vs. 48.3%), suggesting a possible role in increasing susceptibility to CRC. Conversely, the T allele appeared to have a protective effect (37.3% vs. 51.7%). Regarding the G894T polymorphism, the T allele was significantly overrepresented in CRC patients compared to controls (63.3% vs. 26.7%), while the G allele was more frequent in the control group, indicating a protective association (36.7% vs. 73.3%).

To explore the genetic effect of these polymorphisms, dominant and recessive models were evaluated. According to the dominant model (CC + TC vs. TT) for the −786T>C polymorphism, carriers of the C allele showed a higher risk of CRC than homozygotes TT individuals. Similarly, for the G894T polymorphism, individuals carrying the T allele (TT + GT) showed increased susceptibility to CRC compared to those with the GG genotype, confirming the potential role of the T allele in CRC development.

Moreover, the C allele of the −786T>C polymorphism was more frequent in CRC patients than in controls (62.7% vs. 48.3%), suggesting a possible role in increasing CRC susceptibility. Conversely, the T allele appeared to have a protective effect (37.3% vs. 51.7%). For the G894T polymorphism, the T allele was significantly overrepresented in CRC patients compared with controls (63.3% vs. 26.7%), whereas the G allele was more prevalent in the control group, indicating a protective association (36.7% vs. 73.3%). To explore the genetic effect of these polymorphisms, dominant and recessive models were evaluated. Under the dominant model (CC + TC vs. TT) for the −786T>C polymorphism, carriers of the C allele showed a higher risk of CRC compared with TT homozygotes. Similarly, for the G894T polymorphism, individuals carrying the T allele (TT + GT) exhibited an increased susceptibility to CRC compared with those with the GG genotype, confirming the potential role of the T allele in CRC development.

To evaluate whether the associations observed were independent of potential confounding factors, multivariate logistic regression analyses were performed adjusting for age, sex, and BMI. The results demonstrated that the eNOS −786T>C CC genotype remained significantly associated with an increased risk of colorectal cancer (aOR = 2.08, 95% CI: 1.15–3.76, *p* = 0.012), whereas the TT genotype exhibited a protective effect. Similarly, the TT genotype of the G894T polymorphism was independently associated with a markedly increased susceptibility to colorectal cancer (aOR = 8.42, 95% CI: 3.95–14.91, *p* < 0.001). No significant association was observed for the heterozygous genotypes. These findings indicate that the identified genetic variants are independent risk factors for colorectal cancer in the Algerian population (Table 3).

**Figure 3:**
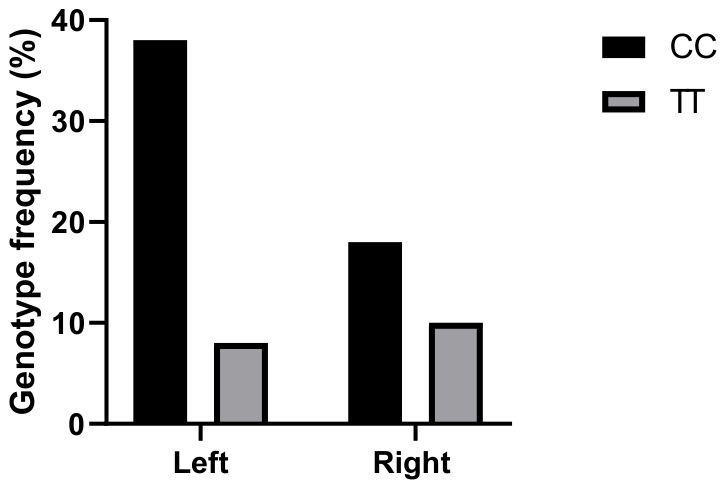
Genotype frequencies of eNOS T-786C polymorphism in CRC patients (N = 150) according to tumor location.

**Figure 4:**
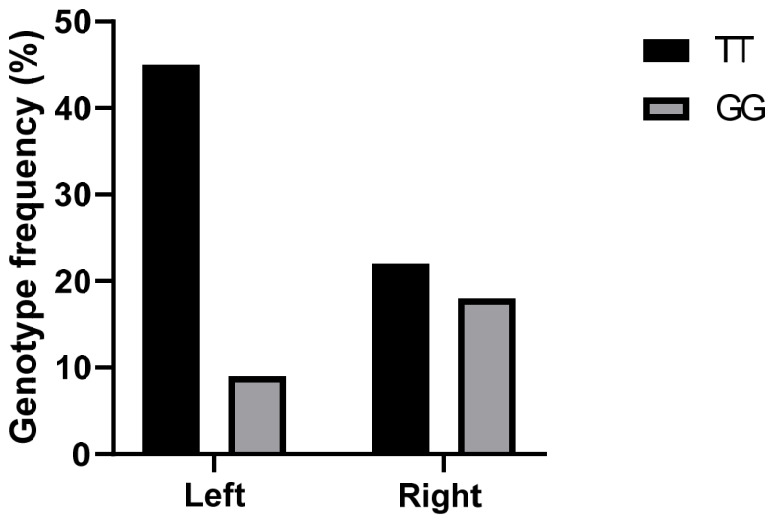
Genotype frequencies of eNOS G894T polymorphism in CRC patients (N = 150) according to tumor location.

Furthermore, the distribution of eNOS polymorphisms differed significantly according to tumor location, with a higher frequency observed in left-sided colorectal cancers compared to right -sided tumors (T-786C: 67.85% vs. 32.14%; G894T: 67.16% vs. 32.83%). Moreover, carriers of the C and T alleles of the NOS3 gene exhibited an approximately threefold increased risk of developing right-sided colon tumors than a left-sided colon tumors (T-786C: OR = 4.46, 95% CI: 1.88–10.65, *p* = 1.5 10^−4^; G894T: OR = 4.18, 95% CI: 1.92–9.18, *p* = 6.3 10^−5^) (Figure 3 and Figure 4).

## 3. Discussion

In this study, we evaluated the association between eNOS polymorphisms (−786T>C and G894T) and susceptibility to colorectal cancer. Our findings showed that the −786 CC genotype was significantly more frequent in CRC patients than in controls and was associated with an increased risk of CRC, while the TT genotype appeared to have a protective effect. Similarly, for the G894T polymorphism, the TT genotype was significantly more frequent in patients, while the GG genotype was more frequent in controls, suggesting a protective association

The endothelial nitric oxide synthase (eNOS/NOS3) gene encodes an enzyme responsible for the production of nitric oxide (NO) from L-arginine, a key signaling molecule involved in vascular homeostasis, immune regulation, and cell signaling pathways [29,30]. Nitric oxide synthases constitute a family of enzymes that catalyze NO synthesis and play a crucial role in the regulation of vascular tone, angiogenesis, and intracellular signaling [31]. Increasing evidence suggests that alterations in NO production may contribute to tumor development and progression, particularly by stimulating angiogenesis, cell proliferation, and metastasis [32].

Nitric oxide plays a complex and dual role in cancer biology, its effects depending on its concentration and the cellular context. At low concentrations, NO promotes angiogenesis and tumor growth, whereas at high concentrations, it exerts cytotoxic and antitumor effects, including the induction of apoptosis and the inhibition of tumor progression. In colorectal cancer, NO has been shown to modulate the tumor microenvironment, promote metastasis, and influence immune evasion mechanisms [33,34,35,36,37]. Therefore, genetic variations affecting NO production could contribute to interindividual differences in susceptibility to CRC.

The −786T>C polymorphism, located in the promoter region of the NOS3 (eNOS) gene, has been shown to reduce transcriptional activity and decrease eNOS expression, thereby altering nitric oxide bioavailability [38]. These alterations could contribute to endothelial dysfunction and promote carcinogenic processes. Similarly, the G894T (Glu298Asp) polymorphism results in an amino acid substitution that can affect the structure and enzymatic activity of the eNOS protein, leading to impaired NO production [39,40].

Previous studies have also reported associations between eNOS polymorphisms and cancer susceptibility. In this context, a case–control study has demonstrated that individuals carrying the T allele of the G894T polymorphism had a higher risk of colorectal cancer than those carrying the G allele [41]. Furthermore, recent meta-analysis studies have suggested that the eNOS 894G>T variant may increase overall cancer susceptibility, thus supporting the potential involvement of this polymorphism in carcinogenesis [42]. However, the results obtained across populations remain heterogeneous, possibly due to differences in ethnicity, environmental exposure, lifestyle factors, and study methodology.

Nitric oxide signaling interacts with multiple molecular pathways involved in colorectal carcinogenesis, including oxidative stress, DNA damage, angiogenesis, and inflammatory responses [43,44,45]. Several authors have indicated that NO plays a key role in regulating tumor progression, immune responses, and the tumor microenvironment in colorectal cancer [46,47,48]. Indeed, recent studies have highlighted the importance of nitric oxide– related signaling pathways, particularly members of the NOS gene family, in modulating these biological processes and their influence on the development of CRC [49,50]. Therefore, genetic variants of NOS genes, including eNOS, could contribute to colorectal carcinogenesis through their impact on NO-mediated signaling pathways.

Interestingly, our results demonstrated a significant association between eNOS polymorphisms and tumor location, with a higher overall frequency of the −786T>C and G894T variants in left-sided colorectal cancers, while carriers of the C and T had a significantly increased risk of developing right-sided tumors. Multivariate logistic regression analyses were conducted to adjust for potential confounding variables, including age, sex, and BMI. The significant associations between eNOS polymorphisms and colorectal cancer risk remained after adjustment, indicating that these genetic variants independently contribute to disease susceptibility. This underscores the importance of nitric oxide–related genetic alterations in colorectal carcinogenesis and highlights their potential utility as predictive biomarkers. This finding highlights the potential role of NOS3 genetic variability in tumor heterogeneity and anatomical distribution.

Although few studies have directly evaluated NOS3 polymorphisms according to CRC laterality, our findings are supported by recent data indicating that nitric oxide-related genetic pathways may differ between right- and left-sided tumors. In this context, a recent study has reported that a NOS2 polymorphism was significantly more frequent in right-sided than in left-sided CRC, suggesting that genetic variations in the nitric oxide pathway contribute to tumor localization [51].

Indeed, right- and left-sided colorectal cancers differ in their embryological origin, molecular profile, and tumor microenvironment [52]. Right-sided colon tumors are typically associated with higher levels of inflammation, oxidative stress, and immune activation, processes in which nitric oxide (NO) plays a key regulatory role [53]. Dysregulated NO production can promote DNA damage, angiogenesis, and tumor progression, thereby contributing to location-specific colorectal carcinogenesis [54].

Furthermore, previous genetic studies have shown that eNOS polymorphisms, particularly G894T, are associated with increased CRC susceptibility, with the T allele linked to a higher cancer risk, although the results remain heterogeneous across populations. Meta-analyses have also emphasized that the association between NOS3 variants and CRC is inconsistent, potentially due to differences in ethnicity, environmental exposure, and methodology. Importantly, most of these studies did not stratify patients according to tumor location, which may partly explain the variability in reported results.

Collectively, our findings provide novel evidence suggesting that eNOS polymorphisms could influence not only susceptibility to colorectal cancer but also tumor localization. The stronger association of the C and T alleles with right-sided colon tumors supports the hypothesis that nitric oxide signaling plays a differential role depending on tumor location. However, large-scale studies and further functional analyses are required to confirm these observations and elucidate the underlying biological mechanisms.

## 4. Materials and Methods

### 4.1. Study Subjects

A total of 150 subjects with CRC followed at Algiers Hospital, as well as 120 unrelated control subjects, were recruited after obtaining their informed consent, in accordance with the Declaration of Helsinki (1964). This study has been approved by the Ethics Committee, the “Algerian National Agency for Research in Health Sciences, ATRSS ex-ANDRS”, in compliance with the Helsinki declaration (Code number 58-DFPR-ATRSS-AAP-2018, approval date: 29 April 2018). An informed consent form was signed by each participant.

### 4.2. Genetic Analyses

Genomic DNA was extracted from peripheral blood leukocytes using the PureLink™ Genomic DNA Mini Kit (Thermo Fisher Scientific, Waltham, MA, USA) according to the manufacturer’s instructions. Genetic variants were analyzed using the polymerase chain reaction–restriction fragment length polymorphism (PCR–RFLP) method with genomic DNA as the template. The −786T>C polymorphism was amplified using the following primers: forward 5′-ATG CTC CCA CCA GGG CAT CA-3′ and reverse 5′-GTC CTT GAA TCT GAC ATT AGG G-3′. PCR amplification was performed for 35 cycles under the following conditions: initial denaturation at 94 °C for 30 s, annealing at 51 °C for 40 s, and extension at 72 °C for 40 s. The amplified PCR products were digested with the restriction enzyme NgOMIV (New England Biolabs, Beverly, MA, USA) at 37 °C for 16 h, and the resulting fragments were separated by agarose gel electrophoresis. The 894G>T polymorphism was amplified using the primers forward 5′-CAT GAG GCT CAG CCC CAG AAC-3′ and reverse 5′-AGT CAA TCC CTT TGG TGC TCA C-3′. PCR amplification was carried out for 35 cycles at 95 °C for 45 s, 63 °C for 45 s, and 72 °C for 45 s. The PCR products were subsequently digested with the restriction enzyme BanII (New England Biolabs, Beverly, MA, USA) at 37 °C for 16 h, and the digestion products were analyzed by agarose gel electrophoresis.

### 4.3. Statistical Analysis

Statistical analyses were performed using GraphPad Prism version 8.0 (GraphPad Software, San Diego, CA, USA). Differences in genotype and allele frequencies between colorectal cancer patients and controls were evaluated using the Chi-square (χ^2^) test. The Hardy–Weinberg equilibrium (HWE) for genotype distributions was assessed in the control group using the Chi-square test. The strength of the association between eNOS polymorphisms and colorectal cancer risk was estimated by calculating odds ratios (ORs) and 95% confidence intervals (CIs). Bonferroni correction was applied to adjust for multiple comparisons among genotypes. Multivariate logistic regression analyses were performed using colorectal cancer status as the dependent variable. Age, sex, and body mass index (BMI) were included as covariates. Adjusted odds ratios (aORs) and 95% confidence intervals (CIs) were calculated to evaluate the independent association between eNOS polymorphisms and colorectal cancer susceptibility. A *p*-value < 0.05 was considered statistically significant.

## 5. Conclusions

Our results suggest that the eNOS −786T>C and G894T polymorphisms are associated with colorectal cancer susceptibility. These findings support the hypothesis that nitric oxide signaling pathways contribute to colorectal carcinogenesis and indicate that eNOS genetic variants could serve as potential biomarkers for CRC risk assessment. Future research integrating genetic, molecular, and environmental factors will be essential to better understand the role of nitric oxide signaling pathways in colorectal cancer development.

## 6. Limitations of This Study

Despite the significant findings, the relatively moderate sample size may limit the statistical power to detect weaker genetic associations and may affect the generalizability of the results to broader populations. Therefore, larger and multicenter studies are warranted to confirm these findings. The case–control design is inherently subject to potential selection and recall biases and does not permit the establishment of causal relationships. Consequently, prospective cohort studies are required to validate the observed associations.

## Figures and Tables

**Table 1:** Clinical data of patients and control.

Parameters	CRC (N = 150)	Control (N = 120)
***Gender***		
Male	77	62
Female	73	58
***Age (y)***	62 ± 13.21	60 ± 10.05
***BMI***	29.03 ± 0.94	28.6 ± 1.20
***Primary tumor localization (%)***		
Left colon	92	-
Right colon	58	-
***TNM stage (%)***		
I + II	35	-
III + IV	110	-
***Tumor size (%)***		
<5 cm	54	-
≥5 cm	96	-

**Table 2:** Genotype frequencies of eNOS in patients and controls.

Genotypes	Patient (N = 150)	Control (N = 120)	*p*	OR 95% CI
**T-786C**				
**CC**	**56(37.33%)**	**26 (21.67%)**	**0.004**	**2.15 (1.21–3.88)**
**TC**	76 (50.67%)	64 (53.33%)	0.37	0.90 (0.54–1.49)
**TT**	**18 (12%)**	**30 (25%)**	**0.005**	**0.41 (0.20–0.81)**
**G894T**				
**TT**	**67 (44.67%)**	**10 (8.33%)**	**7.7 × 10^−12^**	**8.88 (4.19–15.40)**
**GT**	56 (37.33%)	44 (36.67%)	0.50	1.03 (0.62–1.70)
**GG**	**27 (18%)**	**66 (55%)**	**1.7 × 10^−10^**	**0.18 (0.10–0.32)**

**Table 3:** Multivariate Logistic Regression Models.

Genotype	OR (95% CI)	*p*-Value	Adjusted OR (95% CI)	*p*-Value
**Model 1: Association between eNOS T-786C Polymorphism and CRC Risk**				
TT (Reference)	1.00	-	1.00	-
TC	0.90 (0.54–1.49)	0.37	0.92 (0.55–1.53)	0.41
CC	2.15 (1.21–3.88)	0.004	2.08 (1.15–3.76)	0.012
**Model 2: Association between eNOS G894T Polymorphism and CRC Risk**				
GG (Reference)	1.00	-	1.00	-
GT	1.03 (0.62–1.70)	0.50	1.05 (0.63–1.75)	0.47
TT	8.88 (4.19–15.40)	<0.001	8.42 (3.95–14.91)	<0.001
**Model 3: Dominant Genetic Models**				
T-786C: CC + TC vs. TT	1.76 (1.01–3.06)	-	1.71 (0.98–2.98)	0.058
G894T: TT + GT vs. GG	4.47 (2.66–7.53)	-	4.32 (2.55–7.32)	<0.001

## Data Availability

The data presented in this study are available on request from the corresponding author.
